# Should Response-Adapted Therapy Now Be the Standard of Care for Advanced Hodgkin’s Lymphoma?

**DOI:** 10.1007/s11864-017-0460-6

**Published:** 2017-03-13

**Authors:** Peter Johnson, Jemma Longley

**Affiliations:** 1 Cancer Research UK Centre, University of Southampton, Somers Cancer Research Building, Southampton General Hospital, Tremona Rd., Southampton, SO16 6YD UK; 2 Department of Medical Oncology, University Hospital Southampton NHS Foundation Trust, Tremona Rd., Southampton, SO16 6YD UK

**Keywords:** Hodgkin’s lymphoma, FDG-PET, Chemotherapy-related toxicity, Response-adapted therapy

## Abstract

The choice of treatment for advanced Hodgkin’s lymphoma has traditionally been made using an assessment of the baseline risk factors and a judgement of the balance between efficacy and toxicity for the group in question. The use of functional imaging with 2-(18F)-fluoro-2-deoxy-D-glucose (FDG)-positron emission tomography (PET) early in the course of therapy offers a way to make treatment better adjusted to the most important feature of Hodgkin’s lymphoma: the response to therapy. Recent studies have shown that excellent results can be achieved by using early FDG-PET to modulate therapy, with escalation for those with an unsatisfactory response and treatment reduction for those with the most chemosensitive disease. The results of these trials indicate that response-adapted therapy should now become part of the standard approach to care, offering opportunities to improve the results further by indicating those subgroups in need of new approaches such as the emerging antibody-based treatments.

## Introduction

Advances in the treatment of Hodgkin’s lymphoma (HL) with doxorubicin, bleomycin, vinblastine and dacarbazine (ABVD) and escalated treatment with more intensive regimens such as bleomycin, etoposide, doxorubicin, cyclophosphamide, vincristine, procarbazine and prednisolone (BEACOPP) have led to high cure rates and long-term survivorship [1]. This is offset by the risks of short-term and long-term toxicity in those survivors, such as second malignancies, infertility, pulmonary and cardiac problems often exacerbated by radiotherapy, especially in those receiving more intensive regimens. Escalated BEACOPP (eBEACCOPP) results in improved tumour responsiveness by 10–15 percentage points when compared to ABVD in randomised studies, but much less difference in overall survival (OS), mainly due to the majority of patients with relapsed disease being effectively re-treated following first-line ABVD, and differences in treatment-related mortality [2]. Identifying patients with high-risk disease and a poor response to treatment early on promises a more individualised approach, restricting the risk of greater long-term toxicity to those patients who stand to gain maximum survival benefit from intensified therapy.

## Toxicity associated with current treatment approaches

The aim of using a response-adapted approach is to minimise avoidable toxicity while optimising cure rates. Second epithelial cancers are a leading cause of mortality among long-term HL survivors [3]. The increased incidence of breast cancer among women compared to the general population is well documented, with the largest relative risk (from 60-fold to 112-fold) reported for patients treated at age 16 years or younger [4]. Higher doses of radiotherapy and larger volumes of irradiation to breast tissue also increase the risk. The use of both radiotherapy and alkalating chemotherapy increases the risk of lung cancer in HL survivors. This is directly related to higher radiation dosage, where there is a 7-fold to 9-fold increase in patients receiving doses >30 Gy compared with <5 Gy [5]. The overall survival of these patients is significantly lower compared to other patients with lung cancer, possibly due to prior radiation exposure limiting treatment options or a more aggressive form of the disease induced by radiation.

The development of coronary artery disease (CHD) as a consequence of mediastinal radiotherapy correlates with dosage, with a 2.5-fold increase risk in those patients receiving >20 Gy to the mediastium compared with no radiotherapy [6]. As there does not appear to be a safe lower limit of radiotherapy protecting against the development of CHD, it is important to identify those patients likely to be cured by chemotherapy alone early in the treatment course, thereby reducing the number of patients exposed to long-term cardiopulmonary toxicity. The recognition and treatment of CHD risk factors at diagnosis and increased physical activity with maintaining a healthly lifestyle in HL survivors also reduce risk [6].

Infertility is more common among long-term HL survivors and correlates with the use of more intensive chemotherapy regimes, with up to one third of women over 30 experiencing severe menopausal symptoms after treatment with BEACOPP in the German Hodgkin Study Group (GHSG) HD13 and HD15 trials. In men who received 6 to 8 cycles of BEACOPP, 89% had FSH levels consistent with oligospermia. [7]. In contrast, ABVD is less toxic, with the majority of young patients retaining their fertility with a negligible risk of secondary malignancy or leukaemia with this regimen alone [8].

Bleomycin is known to cause pnuemonitis in a small proportion of patients, especially those receiving consolidation radiotherapy over the age of 40, leading to long-term pulmonary toxicity [9]. A retrospective analysis over a 10-year period between 1999 and 2008 by the GHSG in 2015 found that discontinuation of bleomycin during the course of BEACOPP chemotherapy did not alter 5-year overall survival (OS) or progression-free survival (PFS) when comparing groups receiving 4 cycles or less with more than 4 cycles of bleomycin [9]. Similar findings have been reported in other retrospective studies in small subgroups of patients who did not receive bleomycin due to respiratory co-morbidity, leading to the hypothesis that this drug may be safely omitted in other regimens such as ABVD, in patients with favourable disease whose risks of long-term pulmonary toxicity outweigh the benefits of initial response to treatment [10]. Studies in early stage favourable HL by the GHSG however showed that omission of bleomycin from ABVD altogether reduced PFS in this cohort of patients, indicating that the addition of bleomycin early on in treatment, espcecially in patients with high-risk advanced disease, may be important to optimise results [11].

The development of treatment-related acute myeloid leukaemia and myelodysplastic syndrome (tAML/tMDS) has a poor prognosis and typically occurs between 2 and 8 years following initial treatment for HL [12]. Rising cumulative exposure to topoisomerase II inhibitors or alkylating agents such as etoposide and cyclophosphamide is associated with increased risk owing to their effects on DNA replication. This was demonstrated by the GHSG, that showed a cumulative incidence of 1.7% over 6 years in patients receiving more than 4 cycles of BEACOPP, compared with 0.7% of patients receiving less than 4 cycles or none [12].

## Prognostic tools in Hodgkin’s lymphoma

The International Prognostic Score (IPS) is the most widely used risk stratification tool when planning treatment strategies for advanced HL. Seven independent risk factors associated with poor outcome were identified in 5000 patients all treated before 1990. Increased survival rates resulting from treatment advances with newer regimens are not reflected in this scoring system, leading to a more recent re-analysis by the British Columbia Cancer Agency. This showed that while the IPS still gives valuable information about prognosis, its level of discrimination for poor risk groups is reduced; therefore, decision making regarding allocation of therapy using baseline characteristics is less reliable [13]. The recent development of gene expression profiling techniques may help physicians plan initial treatment strategies for HL, by identifying patients in high-risk groups for relapse when treated with less intensive regimens. The prognostic value of a 23-gene classifier has been described by Scott et al. [14], who found this superior to the IPS score and immunohistochemistry, with a 5-year OS of 63% versus 92% in the high-risk and low-risk groups respectively, although other groups have yet to replicate these findings. Elevation of specific cytokines including soluble CD30 and CCL17 (TARC) has been shown in small retrospective studies to be related to advanced disease and poorer outcomes in HL. Monitoring of disease response by measuring these potential peripheral blood biomarkers is an attractive option when planning and adapting treatment, but larger prospective trials are needed to validate their use [15, 16].

Early response to treatment seems to be the most important prognostic factor in HL. Patients where first-line treatment fails to induce complete remission have a poorer prognosis and need to be identified as early as possible in order to modify treatment accordingly. The use of conventional computerised tomography (CT) to monitor disease activity is unsatisfactory, owing to its limited ability to distinguish between normal and abnormal lymph nodes based on size alone, with no information regarding cellular function [17]. The initial staging of disease by anatomical extent is also imperfect, risking both over-treatment and under-treatment. Positron emission tomography (PET) with 2-(18F)-fluoro-2-deoxy-D-glucose (FDG) when combined with CT has been shown effective in identifying metabolically active tissue at sites of HL involvement [18]. Retrospective analyses showed that PET-CT following 2 cycles of chemotherapy (PET-2) had a high positive predictive value with a 2-year PFS of 12.8% in PET-2-positive patients, compared to 95% in PET-2-negative patients [17]. This held true for patients independent of IPS score, with patients treated with ABVD in low-risk IPS groups and a positive PET-2 scan at equally high risk of treatment failure as those in high IPS groups [17, 19].

**Fig. 1:**
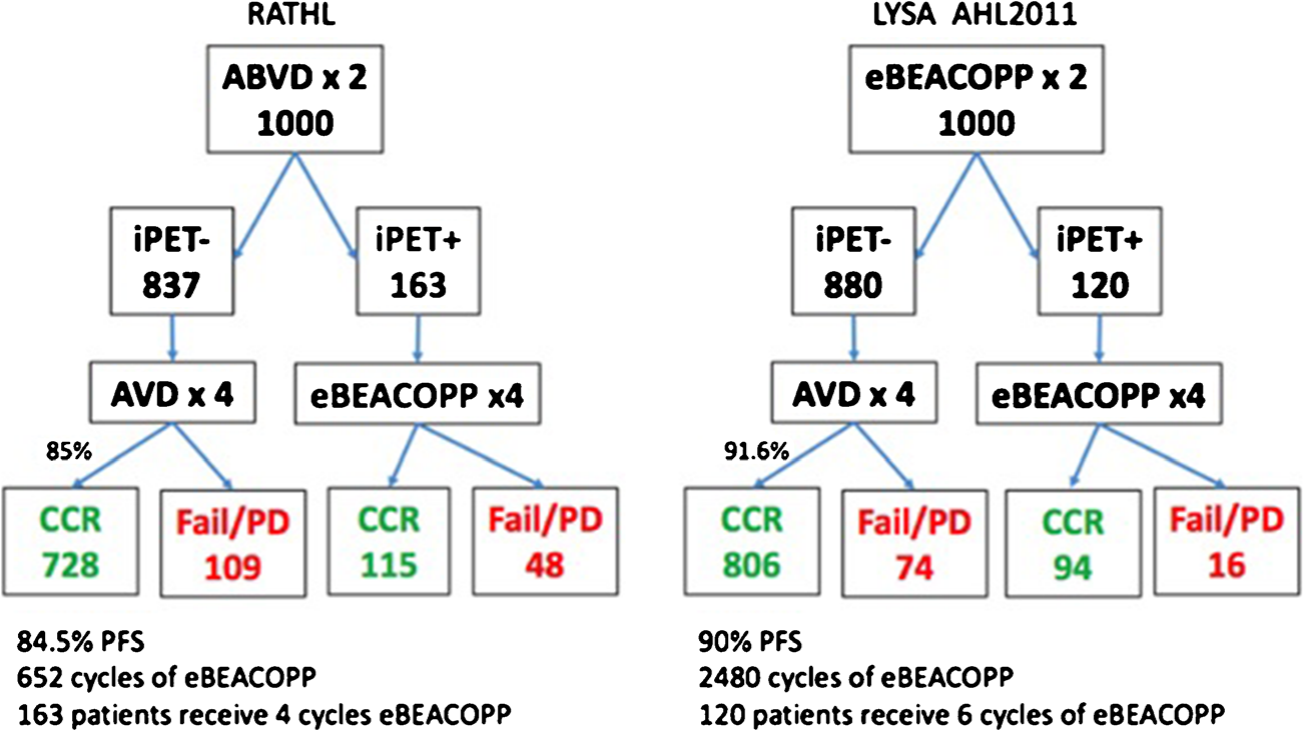
Comparison of initial treatment approaches in RATHL and LYSA trials.

Accurate and reproducible interpretation of PET-CT is fundamental when using this information to guide a response-adapted approach to treatment. Qualitative visual scan interpretation by readers can result in unacceptable variability. This has led to the introduction of a semi-quantitative assessment of PET-CT using the five-point (‘Deauville’) score which has proven broadly reproducible in prospective comparative analyses [20]. The scale allows the cutpoint to be modulated according to trial design, but scores of 1–3 are generally reported as ‘negative’ and 4–5 as ‘positive’ (see Table 1 and Fig. 1). This has been validated in a large international study with six independent blinded reporters who scored PET-CT scans at baseline and following 2 cycles of ABVD (PET-2) using the five-point scoring system [20]. The positive and negative predictive values of PET-2 reflected results from previous studies, with a 3-year PFS of 95% in PET-2-negative patients versus 13% in PET-2-positive patients, and confirmed the limited role of IPS in identifying patients at risk of treatment failure [21]. Consensus among reviewers in evaluating whether PET-2 was positive or negative was 82% when using the five-point scale [21], with differences in identifying sites of disease and misinterpretation in the physiological uptake of FDG in brown fat, the gut and blood vessels.

**Table 1:** Deauville five-point scoring system

1	No uptake
2	Uptake less than or equal to mediastinum
3	Uptake greater than mediastinum but less than or equal to liver
4	Moderately increased uptake compared with liver
5	Markedly increased uptake compared with liver and/or new lesions
X	New areas of uptake unlikely to be related to lymphoma

## Evidence supporting interim PET-directed therapy

Response-adapted therapy in advanced stage HL is the subject of several recent trials, exploring the de-escalation of treatment in those patients with a good outlook and escalation for those at highest risk of treatment failure. Escalation to BEACOPP in patients with PET-2-positive scans following 2 cycles of ABVD in the International Response-Adapted Therapy for Advanced Hodgkin Lymphoma (RATHL) trial, US Intergroup Study and Italian GITIL 0607 study all showed similar OS and PFS rates in this high-risk cohort of patients, with two thirds of patients disease free at 3 years [22••, 23, 24]. In the RATHL trial, treatment failures were most common in patients with a PET-2 Deauville score of 5 (20 patients out of 38). Although this group is small, it suggests a higher risk of relapse in this population of patients, where treatment could be further escalated at this stage to autologus stem cell transplantation (SCT) or newer approaches with antibody-drug conjugates or checkpoint-blocking antibodies, rather than continuing with BEACOPP. A more intensive approach in a phase II Italian study using SCT following ifosfamide/gemcitabine/etoposide/vinblastine (IGEV) salvage therapy in PET-2-positive patients resulted in a 2-year PFS of 76%, compared with 82% in the PET-negative group who received 6 cycles of ABVD, although the definition of PET positive here included those with a score of 3 on the five-point scale [25]. There is a risk that long-term PFS in the SCT group may be offset by the increased difficulty of further therapy and toxicity including development of secondary cancers.

The de-escalation of treatment in PET-2-negative patients has been assessed in both the RATHL trial and French Lymphoma Study Association (LYSA) study. The LYSA study’s initial treatment strategy used 2 cycles of BEACOPP with patients randomised to continue BEACOPP versus de-escalation to ABVD for the remaining 4 cycles, while RATHL initially treated patients with 2 cycles of AVBD, then randomised to continue AVBD versus de-escalation to AVD [22••, 26••]. The omission of bleomycin after 2 cycles of ABVD in the RATHL trial, in the 84% of patients with a negative PET-2, resulted in a lower incidence of pulmonary toxic effects compared with 6 cycles including bleomycin, with a 3-year PFS of 85.7 and 84.4% in the ABVD versus AVD groups respectively and similar 3-year OS in both groups (97.2 versus 97.6%). Longer term follow-up is needed to establish the effect of treatment de-escalation on morbidity and mortality, but early conclusions point to the safe omission of bleomycin in patients with a good initial response to treatment. The use of PET in patients initially treated with BEACOPP resulted in lower PET false negative rates in the LYSA study when compared with the use of ABVD in the RATHL trial [22••, 26••]. Figure 1 uses the reported outcomes from these studies to model a comparison between both approaches. Initial treatment with BEACOPP in patients who became PET-2 negative resulted in less early recurrences, but at the cost of exposing a larger number to 2 cycles of the more intensive treatment. One approach to this finding is for patients with high stage or IPS at diagnosis to receive more intensive regimens such as eBEACOPP, as the false negative rate was higher (20%) in patients with stage 4 disease in the RATHL trial. Patients with lower stage disease and IPS score, where a negative PET scan is prognostically more reliable after ABVD, could start with this regime, to avoid exposing this group to the long-term toxicity of eBEACOPP.

The use of radiotherapy guided by persistent PET-positive masses at the end of treatment with chemotherapy was assessed by the GHSG HD15 trial, where patients with residual masses above 2.5 cm which were PET avid received additional radiotherapy with 30 Gy after 6 cycles of eBEACOPP [27]. The negative predictive value of PET in this context was 94%. The RATHL trial concluded that the omission of consolidation of radiotherapy in patients who had a negative PET-2 with bulky disease and residual masses was a safe approach for low-risk patients, with only 6.5% of patients receiving radiotherapy, compared with 38 to 50% of patients in previous studies with no worsening of 3-year PFS [22••].

Brentuximab vedotin is an anti-CD30 antibody-drug conjugate with proven activity in patients with relapsed HL who have been heaviliy pre-treated. Recent 5-year survival data in patients treated with brentuximab after failed autologous SCT in the phase II trial setting showed that a proportion of patients who had a complete response (CR) remained disease free at 5 years (13/34 patients) with a 5-year OS of 41% within the whole patient population (*n* = 102) [28]. This impressive efficacy as a single agent and high response rate in combination with AVD led to the prospective ECHELON-1 trial comparing brentuximab vedotin and AVD in the first-line setting with AVBD. The phase I/II combination study showed that brentuximab vedotin given with bleomycin results in very severe pulmonary toxicity [29•]. The same risks do not appear to pertain with sequential treatment using brentuximab in patients previously treated with bleomycin, although the safe interval between the two remains to be determined. The combination of brentuximab with more intensive regimens when escalating treatment for PET-2-positive patients is certainly an approach which could be considered but would require careful safety assessment.

Nivolumab and pembrolizumab are both anti-programmed cell death (PD-1) monoclonal antibodies which have shown significant activity in phase I/II trials in HL patients who previously received extensive treatment, including autologus SCT and brentuximab vedotin. Early interim results from phase I/II studies showed an objective response in 66% of patients treated with nivolumab who had recurrent or resistant disease following prior treatment with both autologous SCT and brentuximab vedotin [30•]. Similar results were reported by Armand et al., who showed that heavily pre-treated patients receiving pembrolizumab had an objective response rate of 65%, with 70% maintaining that response at 24 weeks [31]. Longer follow-up is needed to assess the durability of these responses, and overall, about 25% of patients seem to have durable remissions with single-agent therapy, similar to that of single-agent brentuximab vedotin ([30•], 32). Nevertheless, this suggests a promising treatment for this cohort of patients where options are limited, but the challenge remains to identify those patients most likely to benefit in the long term by the development of specific biomarkers, given that PDL1 expression on tumour cells in HL is less predictive of response when compared to solid tumours [31].

Response-adapted therapy allows a more personalised treatment approach, but careful evaluation in large randomised controlled trials as described above is needed to ensure the safety of patients. The use of more intensive regimes such as eBEACOPP in the initial treatment of HL improves the false negative rates of PET-2 in high-risk patients, but treatment still fails in the one third of patients with PET-2-positive scans. There is clearly room for improvement in this cohort, where brentuximab vedotin in combination with chemotherapy or anti-PD-1 therapies might improve efficacy. The use of these therapies in the front-line setting needs to be evaluated in randomised trials, where the interpretation of PET is likely to become more challenging due to the inflammatory responses seen with immunotherapy, increasing the risk of false positives. De-escalation in PET-2-negative groups with the omission of bleomycin and consolidation radiotherapy seems a good approach for reducing toxicity without affecting efficacy in this patient group, although further follow-up is needed to assess the effect on long-term survival.

## Conclusions

The safety and efficacy of a response-adapted approach to the treatment of advanced HL have been demonstrated in recent trials, with advances in knowledge of how to safely de-escalate treatment in lower risk patients and intensify it in those at highest risk of relapse. In those patients with highest risk of treatment failure with more intensive regimes, novel therapies may provide improved efficacy. FDG-PET for is not a perfect test, with important false negative rates in those with high-risk disease treated with less intensive regimens and the theoretical risk of increased false positive rates with immunotherapy. The use of gene expression analyses and other biomarkers to stratify patients may become important in the future as additional means to predict the outcomes of therapy.
